# Preoperative RDW-Based composite score for predicting mortality after hip fracture surgery

**DOI:** 10.1186/s12891-026-09578-3

**Published:** 2026-02-16

**Authors:** Sevim Şenol Karataş, Sait Fatih Öner, Oğuz Kağan Bulut, Hacı Bayram Tosun

**Affiliations:** 1 Department of Anesthesiology, Elazig Fethi Sekin City Hospital, University of Health Sciences, Elazig, Türkiye; 2 Department of Orthopaedics and Traumatology, University of Health Sciences, Elazig Fethi Sekin City Hospital, Elazig, Türkiye

**Keywords:** Elderly patients, Prognosis, Red cell distribution width, Lactate-to-albumin ratio

## Abstract

**Introduction:**

To develop a simple preoperative score based on age, red cell distribution width–standard deviation (RDW-SD), and the lactate-to-albumin ratio (LAR), and to evaluate its performance in predicting 3-month and 1-year mortality after femoral neck fracture treated with primary total hip arthroplasty.

**Materials and methods:**

In this single-center retrospective cohort, 666 consecutive patients who underwent hip fracture surgery between January 2020 and June 2024 were analyzed. The primary outcomes were 3-month and 1-year all-cause mortality. Preoperative demographic characteristics and laboratory parameters (RDW-SD, lactate, albumin, and neutrophil-to-lymphocyte ratio) were recorded. Candidate predictors were screened using univariable logistic regression, followed by a multivariable model including age, RDW-SD, and LAR. An integer risk score was derived from regression coefficients.

**Results:**

Overall, 245 patients (36.8%) died within 1 year. Non-survivors were older and had higher RDW-SD, lactate, LAR, and neutrophil-to-lymphocyte ratio, and lower albumin levels than survivors (all *p* < 0.01). In multivariable analysis, age, RDW-SD, and LAR remained independent predictors and were combined into an age–RDW-SD–LAR score ranging from 0 to 12 points. When classified into three risk groups (0–3, 4–7, and 8–12), observed 1-year mortality rates were 10.7%, 35.6%, and 62.8%, respectively, closely matching model-predicted estimates. The score showed good discrimination for 1-year mortality (AUC 0.78; 95% CI 0.75–0.82) with similar performance after bootstrap internal validation. Albumin was expressed in g/L, and the lactate-to-albumin ratio was calculated accordingly.

**Conclusions:**

The age–RDW-SD–LAR score, based on routinely available preoperative laboratory measures, provides a simple and clinically applicable tool for estimating early and late mortality after femoral neck fracture treated with primary total hip arthroplasty and may support preoperative risk stratification, pending external validation.

## Introduction

Hip fractures represent a major health problem, particularly in older adults, and are associated with substantial mortality. One-year mortality after hip fracture varies across countries and cohorts but is reported to be approximately 20–25% in most series [1]. This high mortality reflects not only complications directly related to the fracture but also multiple contributing factors such as advanced age, comorbidities, and diminished preoperative physiological reserve [2].

Traditional risk assessment tools primarily incorporate variables such as fracture type, timing of surgery, and comorbidity burden. However, there is growing interest in the use of accessible, inexpensive, laboratory-based prognostic markers. In this context, red cell distribution width (RDW), a routinely available parameter obtained from complete blood count testing, has been associated with mortality in various conditions, including cardiovascular disease, infection, and frailty in older adults [3].

Several studies have also demonstrated that elevated RDW increases postoperative mortality in patients with hip fractures. In a study of geriatric patients undergoing hip fracture surgery, a preoperative RDW ≥ 14.25% was associated with a nearly 4.7-fold increase in 30-day mortality and a 2.74-fold increase in 1-year mortality [4]. In recent years, numerous investigations—particularly in critically ill populations—have reported that ratio-based biomarkers such as the lactate-to-albumin ratio (LAR) or RDW-to-albumin ratio may serve as strong predictors of mortality [5].

Despite these findings, few studies have evaluated both RDW and LAR simultaneously in older adults with hip fractures, developed a composite scoring system from these markers, or assessed their combined prognostic value. Existing research has typically focused on single biomarkers, leaving the prognostic potential of ratio-based combinations underexplored [6].

Given these gaps, developing an objective, inexpensive, and easily measurable prognostic score based on simple preoperative parameters—such as RDW-SD, LAR, and age—may help clinicians better determine preoperative risk and optimize perioperative care strategies.

The aim of this study was to develop a simple laboratory-based risk score composed of age, RDW-SD, and LAR in older adults undergoing hip fracture surgery, and to evaluate its ability to predict 3-month and 1-year mortality with robust internal validation.

The intended application of this model is limited to patients with femoral neck fractures treated with primary total hip arthroplasty and should not be generalized to all hip fracture types.

## Materials and methods

This single-center retrospective observational cohort study included a total of 820 patients who underwent surgery for hip fracture at our institution between January 2020 and June 2024.

To clarify the study timeline and patient flow, the annual distribution of included cases was as follows: 2020 (*n* = 121), 2021 (*n* = 124), 2022 (*n* = 181), 2023 (*n* = 161), and 2024 (January–June, *n* = 79), yielding a total of *n* = 666.

According to the Oxford Centre for Evidence-Based Medicine classification, this study represents Level III evidence. All patients were followed for mortality outcomes for up to one year after surgery, and follow-up for all included patients was completed prior to ethics committee approval.

### Study population and selection criteria

Inclusion criteria were as follows: (1) adult patients (≥ 18 years) who underwent surgical treatment for hip fracture at our institution during the study period, (2) availability of preoperative laboratory measurements including RDW-SD, lactate, and albumin obtained before surgery, and (3) availability of complete follow-up data for 3-month and 1-year all-cause mortality.

Only low-energy fragility fractures resulting from age-related falls were included in the study. All fractures were femoral neck fractures. Patients with hip fractures due to high-energy trauma, including motor vehicle accidents or other major traumatic mechanisms, were excluded.

All included patients were treated with primary total hip arthroplasty. No patients underwent hemiarthroplasty or internal fixation procedures. Accordingly, heterogeneity related to surgical treatment was minimized in the study cohort.

All preoperative laboratory measurements were obtained within the first 24 h after hospital admission and before surgery, according to the institution’s standardized preoperative assessment protocol. No laboratory values obtained after surgical intervention were included in the analysis.

At our institution, primary total hip arthroplasty is the standard treatment for displaced femoral neck fractures in medically operable patients, regardless of age, based on an institutional care pathway. Patients deemed too frail or unsuitable for THA due to extreme comorbidity burden or limited life expectancy were not included, as they were managed non-operatively or referred to alternative pathways.

### Exclusion criteria

Patients were excluded if they had missing preoperative laboratory data (*n* = 62), unavailable or incomplete 1-year mortality information (*n* = 48), pathological fractures or revision surgery indicating a different clinical context (*n* = 31), or age < 18 years or inconsistent clinical or laboratory records (*n* = 13).

**Fig. 1:**
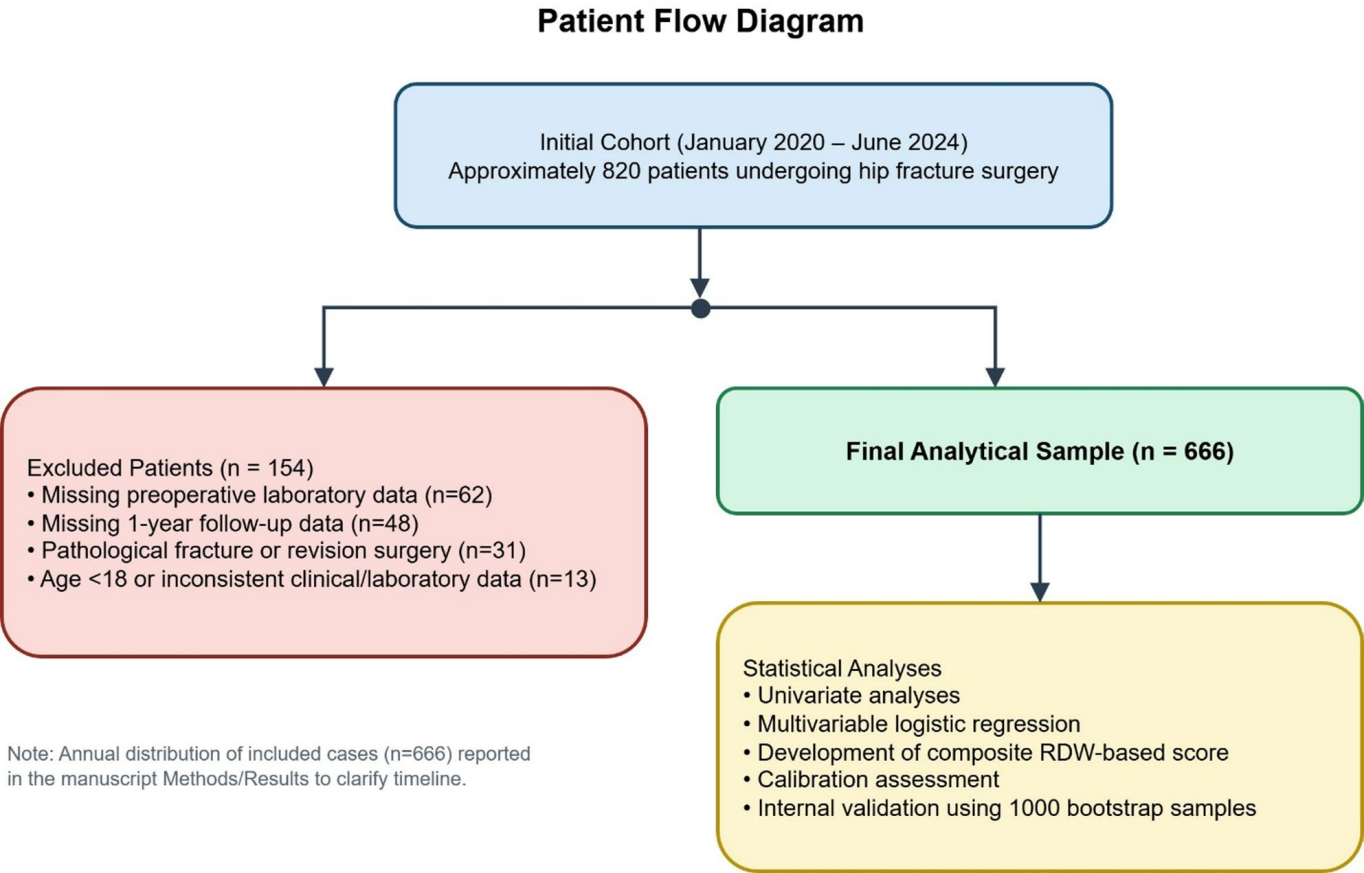
Study flow diagram and analytical framework for the development of the RDW-based composite score

After applying exclusion criteria, 666 patients were included in the final analysis. At 1-year follow-up, 421 patients were alive and 245 had died from all causes. All analyses were conducted using this final cohort (Fig. 1). This retrospective observational cohort study was conducted and reported in accordance with the STROBE (Strengthening the Reporting of Observational Studies in Epidemiology) guideline and the TRIPOD recommendations for prognostic model development, and was approved by the local ethics committee in accordance with the principles of the Declaration of Helsinki.

Demographic characteristics, comorbidities, and laboratory parameters were recorded using a standardized data collection form. Laboratory variables included hemoglobin, RDW-CV, RDW-SD, lactate, albumin, absolute neutrophil and lymphocyte counts, and other routine biochemical parameters.

The lactate-to-albumin ratio (LAR) was calculated by dividing lactate (mmol/L) by albumin (g/L). The neutrophil-to-lymphocyte ratio (NLR) was calculated by dividing the absolute neutrophil count by the lymphocyte count. RDW-CV and RDW-SD values were automatically reported by the complete blood count analyzer. All laboratory measurements were performed in the same hospital laboratory using a single standardized analytical platform throughout the study period, and no major changes in laboratory equipment or measurement protocols occurred during the study interval.

The primary outcome was 1-year all-cause mortality following surgery. The secondary outcome was 3-month all-cause mortality. Mortality outcomes were confirmed using national population registry data and hospital electronic medical records. The prognostic effects of age, RDW-SD, LAR, and other laboratory variables on 3-month and 1-year mortality were evaluated.

### Statistical analysis and model development

The distribution of continuous variables was assessed using the Shapiro–Wilk test and histogram/informal Q–Q plots. Normally distributed variables were presented as mean ± standard deviation, non-normally distributed variables as median (interquartile range), and categorical variables as number (percentage). Between-group comparisons were performed using the Student t-test for normally distributed variables, Mann–Whitney U test for non-normally distributed variables, and chi-square or Fisher’s exact test for categorical variables.

Potential predictors of 3-month and 1-year mortality were first examined using univariable logistic regression, and odds ratios (ORs) with 95% confidence intervals (CIs) were reported. Model development and reporting followed the international TRIPOD recommendations for prognostic studies [7]. In the multivariable model, age, RDW-SD, and LAR were included together. To minimize the risk of overfitting, the core model was restricted to these three predictors, considering the event-per-variable ratio. Continuous variables, including age, RDW-SD, and LAR, were initially evaluated in their continuous form and then categorized based on receiver operating characteristic (ROC) curve analysis, the Youden index, and clinically meaningful thresholds to enhance clinical applicability.

For development of the age–RDW-SD–LAR risk score, categorized age, RDW-SD, and LAR values were entered simultaneously into the multivariable logistic regression model. For each category, β coefficients and adjusted ORs were obtained. To construct a clinically applicable integer-based scoring system, point values were assigned based on the relative magnitude and statistical significance of adjusted regression coefficients, prioritizing clinical interpretability and parsimony. Categories with non-significant or negative regression coefficients were not assigned positive points in the final score. This yielded a total score ranging from 0 to 12 points, with 0–4 points assigned for age, 0–2 points for RDW-SD, and 0–6 points for LAR. The total score was then stratified into three predefined risk groups: low (0–3), intermediate [4–7], and high [8–12].

The discriminative performance of the score was assessed by calculating the area under the ROC curve (AUC) for 1-year mortality, with 95% CIs obtained using bootstrapping. For comparison, AUC values for age, RDW-SD, and LAR individually were also calculated. Calibration was evaluated using a calibration table and calibration plot comparing observed and predicted mortality across risk groups, and by estimating calibration slope and intercept from a logistic regression model using the logit of predicted probabilities.

To evaluate whether categorization of continuous predictors resulted in information loss, alternative logistic regression models retaining variables in their linear continuous form were tested. These models yielded AUC values closely comparable to the categorical model; however, the categorized model was preferred due to its greater clinical practicality.

Internal validation was performed using 1,000 bootstrap resamples. In each bootstrap sample, the model was refitted, and AUC values were calculated for both the bootstrap sample and the original dataset. The mean difference between these values represented optimism, which was subtracted from the apparent AUC to obtain the optimism-corrected (bootstrap-corrected) AUC. Additionally, the Brier score was calculated to assess overall accuracy. A two-sided p-value < 0.05 was considered statistically significant for all analyses. Formal statistical comparison of AUC values using the DeLong test was not performed.

Regression coefficients were approximately scaled by dividing each β coefficient by 0.3 and rounding to the nearest integer to derive point values, while preserving the relative weight of predictors and ensuring clinical interpretability. This approach resulted in 2 points for β ≈ 0.6, 4 points for β ≈ 1.5, and 6 points for β ≈ 2.0.

### Ethical approval

The study was approved by the institutional ethics committee of our hospital due to its retrospective and non-interventional design (approval date: July 24, 2025; approval No: 2025/13–20). The study was conducted in accordance with the principles of the Declaration of Helsinki. All patient identifiers were kept confidential, and data were anonymized prior to analysis.

## Results

A total of 666 patients were included in the study; among them, 421 (63.2%) were alive at 1-year follow-up, whereas 245 (36.8%) had died. Patients who died within one year were significantly older compared with survivors (82.2 ± 9.4 vs. 74.4 ± 10.6 years; p < 0.001). Sex distribution was similar between groups, with females comprising 62.7% and 63.3% of the survivor and non-survivor groups, respectively (p = 0.952). Hemoglobin levels were higher in survivors (12.3 ± 1.8 vs. 11.8 ± 1.9 g/dL; p = 0.003). RDW-CV values were not associated with 1-year mortality (15.1 ± 6.1 vs. 15.3 ± 1.9; p = 0.616), whereas RDW-SD was significantly higher in non-survivors (47.1 ± 5.5 vs. 45.2 ± 5.3 fL; p < 0.001). Preoperative lactate (1.70 [1.20–2.20] vs. 1.20 [0.80–1.40] mmol/L; p < 0.001) and LAR (0.05 [0.04–0.07] vs. 0.03 [0.02–0.04]; p < 0.001) values were markedly elevated in the non-survivor group, whereas albumin levels were lower (32.8 ± 4.4 vs. 34.9 ± 4.1 g/L; *p* < 0.001). The neutrophil-to-lymphocyte ratio (NLR) was also higher among those who died (7.22 [3.90–12.21] vs. 5.52 [3.04–10.22]; *p* = 0.001) (Table 1).

**Table 1:** Baseline demographic and laboratory characteristics of patients according to 1-year survival status

Variable	Survivors (*n* = 421)	Non-survivors (*n* = 245)	*p*-value
Age, years	74.4 ± 10.6	82.2 ± 9.4	< 0.001
Male sex, n (%)	161 (38.2%)	90 (36.7%)	0.72
Hemoglobin, g/dL	12.3 ± 1.8	11.8 ± 1.9	0.003
RDW-CV, %	15.1 ± 6.1	15.3 ± 1.9	0.616
RDW-SD, fL	45.2 ± 5.3	47.1 ± 5.5	< 0.001
Lactate, mmol/L	1.20 (0.80–1.40)	1.70 (1.20–2.20)	< 0.001
Albumin, g/L	34.9 ± 4.1	32.8 ± 4.4	< 0.001
Neutrophils, ×10⁹/L	7.49 (4.96–9.80)	7.62 (5.67–10.43)	0.059
Lymphocytes, ×10⁹/L	1.30 (0.93–1.90)	1.14 (0.75–1.65)	0.002
LAR	0.03 (0.02–0.04)	0.05 (0.04–0.07)	< 0.001
NLR	5.52 (3.04–10.22)	7.22 (3.90–12.21)	0.001

Data are presented as mean ± standard deviation for normally distributed variables or median (interquartile range) for non-normally distributed variables, and as number (percentage) for categorical variables. LAR (lactate-to-albumin ratio) was calculated as lactate (mmol/L) divided by albumin (g/L). NLR = neutrophil-to-lymphocyte ratio

In univariable logistic regression analyses, age, RDW-SD, NLR, LAR×100, and the RDW-SD/RDW-CV ratio were all significantly associated with both 3-month and 1-year mortality (Table 2). Each 1-year increase in age was associated with a 9% increase in 1-year mortality risk (OR: 1.09; 95% CI: 1.07–1.11; p < 0.001). Each 1 fL increase in RDW-SD was independently associated with increased 1-year mortality (OR: 1.07; 95% CI: 1.03–1.10; *p *< 0.001). The OR for LAR×100 was 1.48 (95% CI: 1.36–1.62; p < 0.001), indicating a substantial rise in mortality risk with increasing LAR. NLR was also associated with both 3-month and 1-year mortality, though with a smaller effect size (1-year OR: 1.03; 95% CI: 1.01–1.06; p = 0.005). RDW-CV was not a significant predictor, while the RDW-SD/RDW-CV ratio was associated with 1-year mortality (OR: 1.94; 95% CI: 1.04–3.63; *p* = 0.038).

**Table 2:** Univariable logistic regression analyses for 3-month and 1-year all-cause mortality

Variable	3-month OR (95% CI)	*p*-value	1-year OR (95% CI)	*p*-value
Age (years)	1.08 (1.06–1.11)	< 0.001	1.09 (1.07–1.11)	< 0.001
Male sex (ref: female)	1.04 (0.73–1.47)	0.832	0.99 (0.71–1.37)	0.934
RDW-CV (%)	1.00 (0.97–1.03)	0.945	1.01 (0.98–1.04)	0.693
RDW-SD (fL)	1.05 (1.02–1.08)	0.002	1.07 (1.03–1.10)	< 0.001
NLR (neutrophil-to-lymphocyte ratio)	1.03 (1.00–1.05)	0.028	1.03 (1.01–1.06)	0.005
LAR×100 (lactate [mmol/L]/albumin [g/L] ×100)	1.42 (1.31–1.53)	< 0.001	1.48 (1.36–1.62)	< 0.001
RDW-SD/RDW-CV	2.12 (1.08–4.17)	0.030	1.94 (1.04–3.63)	0.038

Each variable was analyzed in a separate logistic regression model with 3-month or 1-year mortality as the dependent outcome Male sex was compared with females as the reference category

*OR * odds ratio, *CI *confidence interval, *LAR×100* lactate-to-albumin ratio multiplied by 100 to facilitate interpretability

Following TRIPOD recommendations, only strong and clinically interpretable predictors were entered into the multivariable model. The final model included age, RDW-SD, and LAR, with an event-per-variable ratio of approximately 82, indicating a low risk of overfitting. 

The multivariable logistic regression model was constructed using categorized forms of age, RDW-SD, and LAR (Table 3). For age, < 75 years served as the reference; ORs for 75–84 years and ≥ 85 years were 1.91 (95% CI: 1.22–2.98; β = 0.64) and 4.39 (95% CI: 2.73–7.08; β = 1.48), corresponding to 2 and 4 points, respectively. For RDW-SD, compared with < 43 fL, the category ≥ 48.0 fL was associated with an OR of 1.94 (95% CI: 1.19–3.16; β = 0.66) and was assigned 2 points, whereas the intermediate category (43.0–47.9 fL) was not assigned positive points due to lack of statistical significance. For LAR, values of 0.03–0.049 had an OR of 2.43 (95% CI: 1.52–3.87; β = 0.89) corresponding to 3 points, while ≥ 0.05 had an OR of 7.82 (95% CI: 4.87–12.57; β = 2.06) corresponding to 6 points. Thus, the total score derived from age, RDW-SD, and LAR ranged from 0 to 12 (Table 3).

**Table 3:** Derivation of the age–RDW-SD–LAR risk score: variable categories, adjusted odds ratios and assigned points

Variable	Category	β (log-OR)	Adjusted OR (95% CI)	Assigned points
Age	< 75 years	Reference	–	0
	75–84 years	0.64	1.91 (1.22–2.98)	2
	≥ 85 years	1.48	4.39 (2.73–7.08)	4
RDW-SD	< 43 fL	Reference	–	0
	43.0–47.9 fL	−0.05	0.95 (0.61–1.49)	0
	≥ 48.0 fL	0.66	1.94 (1.19–3.16)	2
LAR	< 0.03	Reference	–	0
	0.03–0.049	0.89	2.43 (1.52–3.87)	3
	≥ 0.05	2.06	7.82 (4.87–12.57)	6

Point assignment was based on the relative magnitude and statistical significance of adjusted regression coefficients to ensure clinical interpretability. Categories with non-significant or negative coefficients were not assigned positive points

Patients were categorized into three risk groups: low (0–3 points), intermediate (4–7 points), and high (8–12 points) (Table 4). In the low-risk group (n = 215), observed 3-month and 1-year mortality rates were 7.4% and 10.7%, respectively, while model-predicted rates were 8.2% and 11.7%. Among intermediate-risk patients (n = 225), observed mortality rates were 24.0% at 3 months and 35.6% at 1 year, with predicted values of 23.6% and 32.7%. In the high-risk group (n = 226), observed mortality rates were 52.7% at 3 months and 62.8% at 1 year, whereas predicted values were 52.4% and 64.7%.

**Table 4:** Observed and model-predicted 3-month and 1-year mortality across age–RDW-SD–LAR score risk groups

Risk group	Score range	Number of patients, *n*	Observed 3-month mortality, *n*(%)	Observed 1-year mortality, *n*(%)	Predicted 3-month (%)	Predicted 1-year (%)
Low	0–3	215	16 (7.4)	23 (10.7)	8.2	11.7
Intermediate	4–7	225	54 (24.0)	80 (35.6)	23.6	32.7
High	8–12	226	119 (52.7)	142 (62.8)	52.4	64.7

Risk groups were defined according to total score as follows: low risk (0–3), intermediate risk (4–7), and high risk (8–12)

Predicted mortality probabilities were obtained from the final multivariable logistic regression model using the categorized variables

*RDW-SD* red cell distribution width–standard deviation, *LAR *lactate-to-albumin ratio

**Fig. 2:**
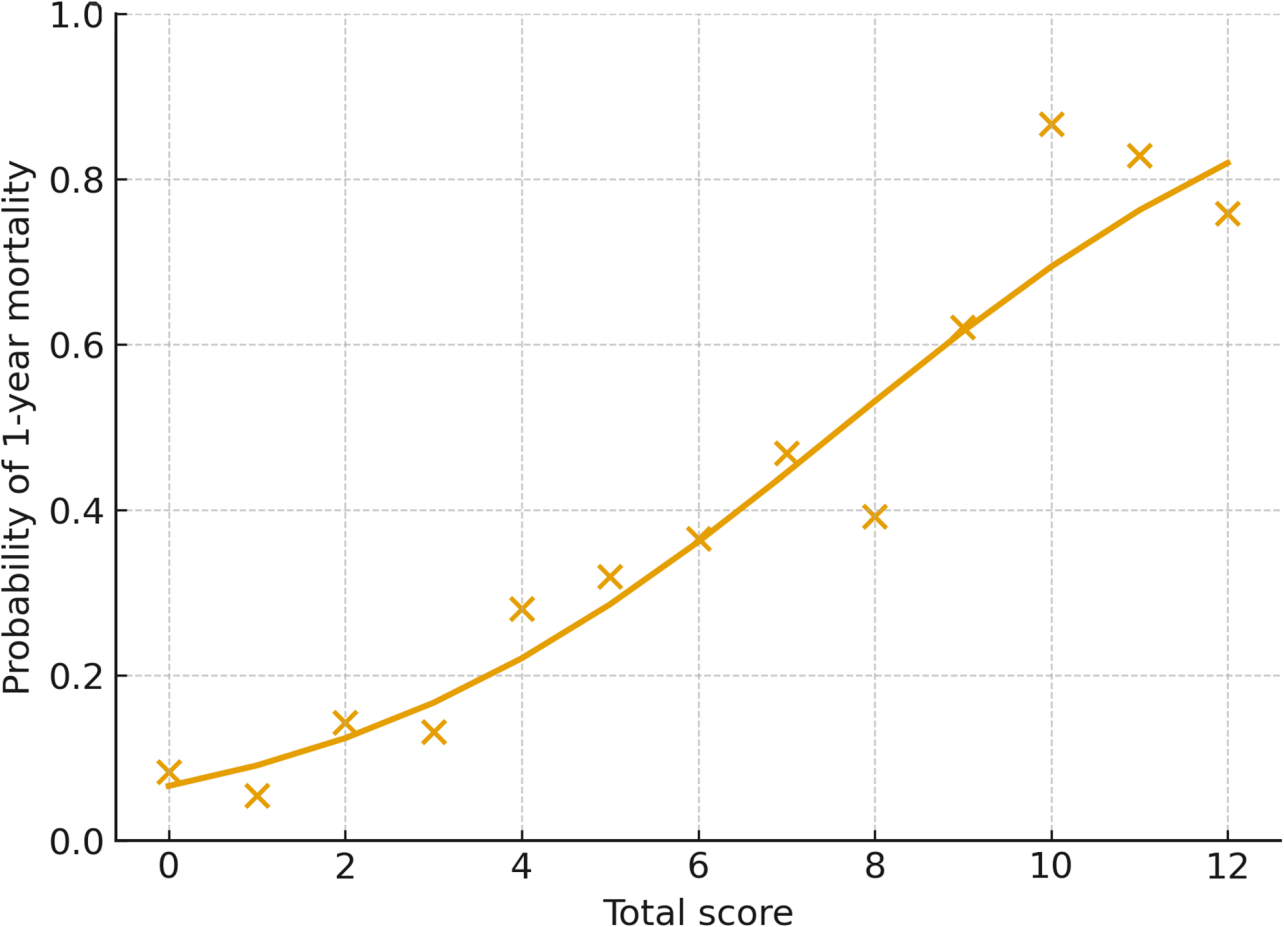
Predicted 1-year mortality probability across the age–RDW-SD–LAR total score. The curve represents model-based predicted mortality probability as a function of the total score, with individual observations shown for illustration

These findings demonstrate that the age–RDW-SD–LAR score provides a clear gradient of risk for both short-term (3 months) and long-term (1 year) mortality. Model calibration was assessed using observed versus predicted mortality across risk strata (Table 4), while Fig. 2 illustrates the model-based predicted mortality probability across the total score.

The discriminative ability of the age–RDW-SD–LAR score for predicting 1-year mortality was good (AUC 0.78; 95% CI: 0.75–0.82). Compared with individual predictors—age (AUC 0.72; 95% CI: 0.67–0.76), RDW-SD (AUC 0.62; 95% CI: 0.57–0.66), and LAR (AUC 0.76; 95% CI: 0.72–0.80)—the combined score yielded a higher AUC. Internal validation via bootstrapping demonstrated a similar optimism-corrected AUC of 0.78, supporting the model’s robustness. The Brier score was 0.18, indicating acceptable overall accuracy in mortality prediction.

## Discussion

In this study, age, RDW-SD, and LAR were identified as independent predictors of 1-year mortality in patients undergoing hip fracture surgery, and the composite age–RDW-SD–LAR score derived from these variables demonstrated effective prediction of both 3-month and 1-year mortality. Although RDW-CV is more commonly reported in routine complete blood counts, RDW-SD was preferred in this study because it reflects the absolute variability in erythrocyte size and is not influenced by mean corpuscular volume. In our cohort, RDW-SD was significantly associated with both short- and long-term mortality, whereas RDW-CV showed no independent association. These findings are consistent with previous studies suggesting that RDW-SD may provide more sensitive prognostic information in heterogeneous clinical settings. Consistent with our findings, a recent multicenter study using multivariable regression analysis demonstrated that advanced age and laboratory-based inflammatory parameters were independently associated with mortality and postoperative complications after hip fracture surgery. These results further support the relevance of integrating readily available clinical and laboratory variables into perioperative risk stratification models [13].

This laboratory-based approach was designed to enable risk estimation even when comprehensive clinical variables required for traditional scores are unavailable at the time of preoperative assessment.

Our findings are consistent with previous research highlighting the prognostic value of hematological and biochemical markers in older adults with hip fractures [8–10].

The composite score achieved an AUC of 0.78 and showed numerically higher AUC values than age, RDW-SD, or LAR alone; however, no formal statistical comparison between AUCs was performed. The minimal optimism observed in bootstrap validation and the calibration slope and intercept values approximating 1.00 and 0.00, respectively, indicate that the model is stable within the cohort and exhibits good calibration. These results align with international reporting recommendations for prediction models, such as the TRIPOD guidelines [7], and emphasize that model performance should be evaluated not only by discrimination metrics but also by calibration and internal validation.

The observed 1-year mortality rate of 36.8% should be interpreted in the context of the advanced age structure of the study population, particularly the high proportion of patients aged ≥ 85 years [11]. Several studies have demonstrated a marked rise in mortality rates among patients aged ≥ 85 years [11, 12]. Our analysis similarly showed a linear increase in mortality risk with age and a substantial elevation in mortality probability among patients aged ≥ 85 years, supporting these earlier observations.

Multiple studies have reported associations between elevated RDW levels and mortality [14, 15]. In patients with hip fractures, RDW has been shown to predict both 30-day and 1-year mortality, with RDW-SD reflecting underlying pathophysiological mechanisms such as inflammation, oxidative stress, and impaired bone marrow function [16]. The persistence of RDW-SD as an independent predictor in our multivariable model suggests that it may provide more sensitive and clinically meaningful prognostic information than RDW-CV.

The strong and independent association between LAR and mortality observed in this study is also noteworthy. The prognostic value of LAR has been extensively examined in critical illness, sepsis, and COVID-19, with studies reporting superior predictive performance compared with lactate or albumin alone [17–19]. Investigations in intensive care settings have shown that LAR is an independent predictor of mortality and achieves higher ROC–AUC values than lactate [20, 21]. However, data regarding the prognostic utility of LAR in hip fracture populations remain limited. Our findings suggest that LAR may serve as an important biomarker in older adults by reflecting both acute metabolic stress and chronic inflammatory and nutritional status.

This study provides a novel contribution to the literature by developing and evaluating a composite risk score based on RDW-SD and LAR for patients with hip fractures. Traditional scoring systems (e.g., NHFS, CCI, POSSUM) require multiple clinical variables and can be time-consuming to calculate [22]. In contrast, the proposed age–RDW-SD–LAR score is simple to compute using routine preoperative laboratory tests and clearly distinguishes mortality risk across low-, intermediate-, and high-risk groups within a 0–12-point range. This demonstrates that clinically meaningful risk stratification can be achieved with minimal data.

Numerous biomarkers have been associated with mortality in hip fracture patients in recent years [23, 24]. However, many of these markers suffer from inconsistent cutoff values, heterogeneous results, or limited feasibility in routine practice. Combining LAR and RDW-SD with age may partially fill this gap by integrating information on inflammatory, hematologic, and metabolic status. The growing use of ratio-based biomarkers in the intensive care literature further supports the applicability of this approach to hip fracture populations [25, 26].

Although associations between RDW, albumin, lactate, and various inflammatory ratios with mortality have been individually reported, no prior study has integrated these parameters into an easy-to-calculate, point-based risk score suitable for bedside use. This study is the first to systematically develop and internally validate a new, simple laboratory-based risk score using RDW-SD, age, and LAR in a large hip fracture cohort (*n* = 666). Additionally, the comprehensive reporting of discrimination, calibration, and bootstrap-validated performance metrics in accordance with TRIPOD strengthens the methodological robustness of the developed score.

The relatively high 1-year mortality observed in this cohort (36.8%) likely reflects the advanced age distribution, the exclusive inclusion of femoral neck fractures, and the referral pattern of our tertiary center, which receives a high proportion of frail and high-risk elderly patients. More than one-third of patients were aged ≥ 85 years, a subgroup known to have substantially higher post-fracture mortality. In addition, the absence of less invasive treatments such as internal fixation or hemiarthroplasty may have contributed to selection of patients with more advanced physiological vulnerability.

### Limitations

This study has several limitations. First, it was conducted at a single center and had a retrospective design; therefore, the generalizability of the findings to other populations may be limited. Although hospital information system records and intensive care databases were carefully reviewed and patients with missing data were excluded, this approach may have introduced selection bias. 

Second, age, RDW-SD, and LAR were initially evaluated as continuous variables; however, to enhance clinical applicability, simplify score calculation, and facilitate interpretation of risk groups, these variables were categorized based on ROC/Youden analyses and clinically meaningful thresholds. This process may lead to loss of information, although the practicality and ease of bedside use offered by the final score mitigate this limitation.

Third, the model was evaluated solely through internal validation, and external validation using an independent cohort from different centers was not feasible. Although the age–RDW-SD–LAR score demonstrated good discrimination and calibration in this single-center cohort, its performance should be confirmed in independent, multicenter populations before routine clinical implementation. Therefore, prospective multicenter studies are needed to confirm the performance of the age–RDW-SD–LAR score. Finally, because the objective of this study was to develop a laboratory-based risk score, direct comparisons with more complex clinical scoring systems such as NHFS or POSSUM were not performed. Future research should investigate how this laboratory-derived score compares with established clinical risk models.

We were unable to directly compare the discrimination and calibration of the proposed score with established clinical risk models (e.g., NHFS, CCI, or an age+ASA model) because key variables required to compute these scores (including ASA class and detailed comorbidity data) were not consistently available in our retrospective dataset. Future prospective and multicenter studies should evaluate the comparative performance of the age–RDW-SD–LAR score against these established models.

Because the age–RDW-SD–LAR score relies exclusively on routinely available preoperative laboratory parameters, it may be readily integrated into standard preoperative assessment workflows to support early risk stratification and perioperative decision-making.The score was developed in a highly selected cohort consisting exclusively of low-energy femoral neck fractures treated with primary total hip arthroplasty; therefore, extrapolation to other hip fracture types or surgical treatments should be avoided.

Fracture displacement classification (e.g., Garden/Pauwels) was not consistently recorded in the retrospective database and could not be analyzed; this may contribute to residual confounding.

## Conclusion

The newly developed score consisting of age, RDW-SD, and LAR demonstrated strong performance in predicting 3-month and 1-year mortality after femoral neck fracture treated with primary total hip arthroplasty. The clear separation of mortality risk across low-, intermediate-, and high-risk categories suggests that the score may have potential clinical utility, although external validation in independent cohorts is required before routine implementation. Although categorizing continuous variables may theoretically result in loss of information, the similar AUC values obtained in alternative models using continuous terms support the rationale for this more clinically implementable categorical structure. Nevertheless, multicenter prospective external validation studies are required to strengthen the generalizability of the model.

These findings should be interpreted as hypothesis-generating, and external validation in independent cohorts is required before routine clinical implementation.

## Data Availability

The datasets used and/or analyzed during the current study are not publicly available due to institutional data protection regulations and patient confidentiality requirements, but are available from the corresponding author on reasonable request.

## Abbreviations

RDW: Red cell distribution width
RDW-SD: Red cell distribution width–standard deviation
RDW-CV: Red cell distribution width–coefficient of variation
LAR: Lactate-to-albumin ratio
NLR: Neutrophil-to-lymphocyte ratio
OR: Odds ratio
CI: Confidence interval
ROC: Receiver operating characteristic
AUC: Area under the curve
